# Evolution from Composome to RNA Replicase

**DOI:** 10.3390/life16020219

**Published:** 2026-01-28

**Authors:** Shaojie Deng, Doron Lancet, Roy Yaniv

**Affiliations:** 1 Chongqing (Fengjie) Municipal Bureau of Planning and Natural Resources, Chongqing 404699, China; 2 Department of Molecular Genetics, Weizmann Institute of Science, Rehovot 7610001, Israel; roy.yaniv@weizmann.ac.il

**Keywords:** origin of life, RNA replicase, RNA hypothesis, metabolism graded autocatalysis replication domain model, stable complex encoding model, compositional information, genetic information, Darwinian evolution, chemical evolution

## Abstract

This paper proposes a novel scheme for the origin of RNA replicase based on the replication-first stable complex evolution (SCE) model, also known as the stable complex encoding (SCE) model, and attempts to derive this scheme from the metabolism-first graded autocatalysis replication domain (GARD) model, thereby theoretically integrating the two hypotheses of the origin of life: replication-first and metabolism-first. Currently, although the replication-first model has made some progress in the artificial selection of RNA replicase, it has yet to achieve a true breakthrough. Meanwhile, metabolism-first models such as the CAS (Collectively Autocatalytic Set) and its graph version RAF (Reflexively Autocatalytic and Food-generated) models, have conducted in-depth research into the origin of metabolic networks but have failed to address the critical transformation issue from metabolism to RNA replication. This paper argues that these two hypotheses should mutually support each other. By introducing oligonucleotide assemblies and expanding the concept of composomes in the GARD model, this paper attempts to understand the general evolutionary mechanism of enzymes, thereby addressing the long-standing neglect of enzymatic catalysis in metabolism-first theories. This integrated scheme not only provides new theoretical support for the evolution of RNA replicase but also offers important insights into solving the key transition problem from chemical evolution to biological evolution.

## 1. Introduction

The exploration of the origin of life remains deeply entrenched in the debate between the two major hypotheses: “metabolism-first” and “RNA (replication)-first.” The former can be traced back to Oparin’s “primordial soup” hypothesis in the 20th century [1], which posits that life began with self-organizing networks of simple organic molecules formed through catalytic processes. The latter, independently advocated by Woese [2], Orgel [3], and Crick [4], suggests that the accidental emergence of the genetic material RNA and the RNA replicase ribozyme was the decisive event in the birth of life. Currently, the replication-first hypothesis holds the majority view, asserting that the earliest self-replicating and evolving entities were informational biopolymers [5,6,7,8]. However, the replication-first model faces seemingly insurmountable challenges. For instance, RNA is considered difficult to synthesize in prebiotic environments and may lack stability, while the theoretically inferred low fidelity of the first RNA replicase would hinder effective information transfer. Consequently, alternative approaches based on early theoretical frameworks have been proposed, such as metabolism-first models centered on autocatalytic sets.

Traditional metabolism-first models often remain at the level of qualitative descriptions. For example, “autocatalytic sets” or “mutually catalytic networks” have abstractly demonstrated the possibility of catalytic closure but fail to incorporate kinetic parameters such as reaction rates and molecular concentrations. This omission renders these models unable to explain how primitive systems maintained constant proportions of components during growth, i.e., achieving a steady state [9]. Thus, although the metabolism-first theory has garnered attention for its alignment with the logic of gradual chemical evolution, it has long been perceived as facing several core difficulties: experimentally, there is a lack of direct observation of steady-state growth in molecular networks; theoretically, it is challenging to quantify how catalytic networks break free from chemical equilibrium to achieve precise regulation akin to modern cellular proliferation [9]. In short, existing evidence is insufficient to prove whether simple molecular networks can achieve self-replication and steady-state maintenance, both of which are core requirements for defining life. Additionally, the metabolism-first theory must address the critique of “Darwinian evolution” [10]—can small molecular networks optimize their structures through natural selection in the absence of genetic material? These unresolved questions highlight the urgent need for breakthroughs in key aspects of the metabolism-first theory to advance its development and validation.

In this context, the research team led by Doron Lancet proposed the “graded autocatalysis replication domain” (GARD) model [11,12], which attempts to construct a mathematically verifiable framework for the metabolism-first theory. For instance, the model introduces two core mechanisms: graded catalytic weights and chemical kinetic simulations [9]. More notably, the GARD model exhibits life-like evolutionary characteristics in computer simulations. When environmental pressures (e.g., scarcity of specific molecules) are introduced, molecular networks of different compositions undergo “natural selection” due to variations in growth efficiency, with highly efficient networks surviving and propagating their compositional information [13,14]. This aligns with the core mechanism of Darwinian evolution. Furthermore, GARD analysis reveals attractor-like transitions from random assemblies to privileged self-organized composomes, confirming that composomes are dissipative systems [14]. Here, composomes can achieve steady-state growth and self-replication. These findings suggest that primitive metabolic systems might have transmitted information through “compositional inheritance” of molecular assemblies rather than nucleic acid sequences, offering a novel perspective on the origin of life: before the emergence of RNA, lipid membrane-encapsulated molecular networks might have already possessed the three fundamental characteristics of life—self-maintenance, steady-state growth, and adaptive evolution. The establishment of the GARD model not only challenges the dominance of the “RNA world” hypothesis but also reshapes scientists’ understanding of the essence of life.

Currently, efforts to validate the replication-first model through artificial selection of RNA replicase have made significant progress but have not yet succeeded. Meanwhile, the metabolism-first model has undergone extensive theoretical exploration but has not provided a pathway from metabolic assemblies to RNA replicase or from compositional information to sequence-based genetics. While these two perspectives appear divergent, it is widely believed that replication-first and metabolism-first hypotheses should not be mutually exclusive [15] but rather mutually supportive. Within the metabolism-first theory, the GARD, CAS and RAF [16] models are prominent and hold promise for integration with the replication-first approach. In particular, the composomes and attractor properties of GARD assemblies could potentially be linked to replication assemblies. It is widely accepted that combining template-replicating biopolymers with mutually catalytic networks would better address the transition from chemical evolution to biological evolution [13]. This paper proposes an origin scheme for RNA replicase based on the replication-first stable complex evolution model (SCE model) [17] and attempts to derive or explain it from the well-studied metabolism-first GARD model. Thus, this work extends the GARD model in some respects while providing theoretical support for the RNA replicase evolution model. Composomes are a key concept in GARD theory, but existing models have only successfully developed lipid composomes. This paper will explain the evolution of RNA replicase from oligonucleotide assemblies based on composomes, addressing the most significant limitation of the metabolism-first theory—the overreliance on non-enzymatic catalysis and the lack of consideration for enzymatic roles.

## 2. From Mutual Catalytic Networks to Replicases

**Figure 1:**
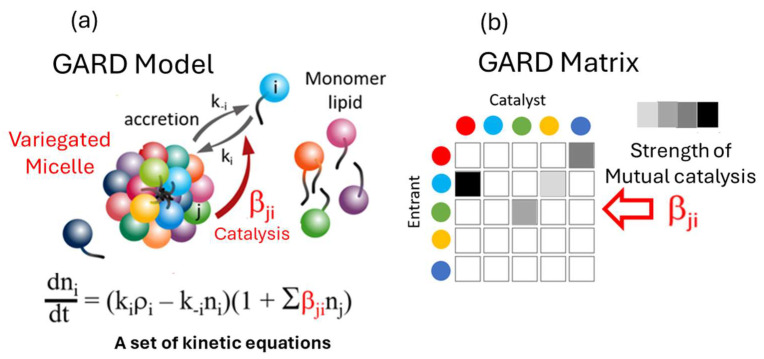
A micelle formed in an environment containing N_G_ different types of lipids (encoded by different colors). (**a**) The kinetics of growth by entry of additional lipid molecules is dictated by the set of N_G_ equations for computing the net rate of entry. (**b**) These rates are controlled by a matrix β of the mutual catalytic values, whereby an element β_ij_ is the catalysis of lipid j in the micelle towards lipid i approaching from the environment.

**Figure 2:**
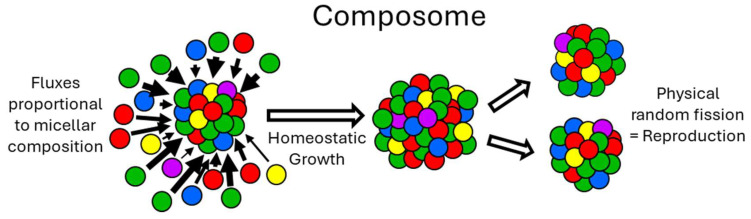
Composome is a micelle with a very specific composition, whereby when computing the rates by the equations of Figure 1, one observes that the ratios among the magnitudes of the influxes is the same ratios among the quantities of molecules of different kinds in the micelle (encoded by different colors). In mathematics, this is described as the flux vector is in the same direction in the N_G_ dimensions. This leads to homeostatic growth, which leads to the formation of two newborns very similar to the parent, i.e., reproduction.

The key transition from chemical evolution to biological evolution lies in the evolution of enzymes, making enzymes the central focus of the origin of life, such as RNA replicases, ribosomes, and proteinaceous metabolic enzymes. Chemical evolution primarily involves non-enzymatic processes, and all theories of autocatalytic sets, including the GARD theory, largely rely on non-enzymatic catalysis. The GARD theory must be expanded to include enzymatic forms, which are related to self-assembled complexes, to demonstrate a plausible pathway from a “lipid-only” environment to what is considered a true protocell [18]. Therefore, it is necessary to explain the evolution of the first RNA replicase or other enzymes within the GARD framework. Generally, it is widely believed that enzyme evolution lacks a specific mechanism and is mainly driven by random mutations. However, general principles of enzyme evolution seem to exist. The SCE model is a theory exploring the fundamental mechanisms of enzyme and functional molecule evolution [17,19] and can be linked to the self-organization, dissipative non-equilibrium steady states, attractors, and self-maintenance of biological systems. Figure 1 and Figure 2 portray composomes in considerable detail.

The SCE model hypothesizes that all functional molecules or enzymes once formed stable complexes with partner molecules through local tight packing, and further evolution and functionality could emerge from these complexes [17,19]. These stable intermolecular interactions are primarily non-covalent and complementary. In other words, an enzyme always forms a stable complex with its substrate before evolving, and then mutations (e.g., sequence changes) lead to catalytic activity. The above description of SCE applies to modern biological systems, where enzymes are primarily peptide chains synthesized from gene sequences and folded in specific ways. However, we can transform it into a more general equivalent form, such as the form of multi-molecular self-assembly or autocatalytic sets: The substrate-bound molecules may form a complex core, and as other molecules sequentially join (accretion), this core is compressed, leading to the formation of catalytic activity. There are experimental examples of catalytic activity generated by aggregate compression strain and specific assembly [20]. This is a general model where any potential substrate or template may be bound by one or more molecules to form catalytic activity, as long as there are appropriate configurations and specific interactions between these molecules. Obviously, functional sequence configurations are very rare and need to be preserved once obtained, which may be the reason for the evolution of genetic coding. Therefore, functional SCE assemblies have specific sequence combinations and form energy-minimized complexes, which is called thermodynamic coding; on the basis of thermodynamic coding, transition-state stabilization and catalytic conversion occur due to the internal specific sequence configuration, which is called kinetic coding. Correspondingly, the stable complex evolution model can also be called the stable complex encoding model [19].

**Figure 3:**
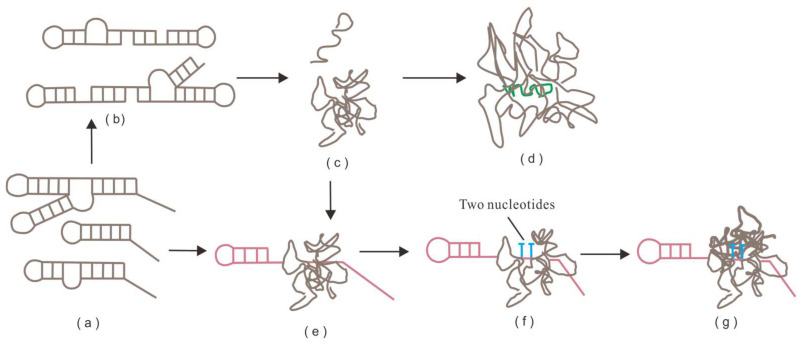
(**a**) RNA can be formed either through abiotic synthesis catalyzed by minerals or under the catalysis of a pure organic system, as shown in Section 5. (**b**) Oligonucleotides form longer and more complex RNAs through hybridization and ligation. (**c**) Single or a few RNAs bound together. (**d**) One or more folded oligonucleotides can bind to any potential substrates (cyan), forming stable complexes. Through accretion, this stable complex will increase in volume and compress the core of the complex, potentially generating catalytic activity towards the potential substrates. The compressive strain of the aggregate may bring atoms into close proximity, leading to catalysis [20]. (**e**) The single-stranded part of the oligonucleotide (red) may be bound by RNA (**f**,**g**). Based on (**e**), if the potential substrate is a single-stranded RNA binding two single nucleotides (blue), then this stable complex may evolve template-dependent RNA replicase activity through accretion (as shown in (**g**)). Since the interactions between the components of the stable oligonucleotide assembly are energy-minimizing, they have high affinity, recognition specificity, and are reproducible. Therefore, the first RNA-dependent RNA replicase does not replicate itself but rather achieves amplification by repeatedly assembling almost identical RNA replicases. Since the RNA replicase with the same composition is formed through repeated assembly, from the perspective of the assembly, the RNA replicase (or the ribozyme binding to any substrate, as shown in (**d**)) is a composome and has compositional information.

The prevailing view suggests that the main difficulties in understanding the evolution of RNA replicase ribozymes are primarily due to the absence of genetic information encoding and evolutionary selection mechanisms, as well as the lack of sufficiently long RNA in the prebiotic chemical environment. However, the evolutionary scheme of RNA replicase proposed by the SCE model can, to a certain extent, circumvent the aforementioned prebiotic chemical and evolutionary mechanism obstacles. It is hypothesized that in an environment where RNA is present, an RNA molecule of a certain length that folds into a three-dimensional conformation binds to a single-stranded RNA (template) and two single nucleotides to form a complex (Figure 3). We note that the specific sequences of the first single-stranded template and nucleotide pairs are unknown because they could be any classic base pairs. According to stereochemistry theory, two pairs of bases inside the assembly can form a phosphodiester bond under specific stereochemical effects without determining the nature of these two pairs of bases. The length and number of oligonucleotides are also unknown, and these two parameters can be coordinately changed. Moreover, the length of oligonucleotides may change significantly through ligation and hybridization.

In this complex, the two nucleotides are adjacent and form complementary base-pairs with the template RNA. This is an SCE assembly containing a special template-nucleotide substrate. Although single nucleotides tend to form complementary base-pairs with the single-stranded RNA, they usually can only form a stable complex together with the assistance of other molecules. This complex serves as the foundation for the evolution of RNA replicase. Its surface can bind multiple small RNAs through accretion, which may predominantly be in a stably folded form [21,22]. The non-covalent self-assembly between these amphiphilic building blocks is mainly driven by hydrophobic interactions, such as base stacking in RNA [23]. Typically, mutations on the surface of the enzyme-substrate complex or the binding of allosteric effector molecules can alter catalytic activity [24,25]. Therefore, the allosteric effect resulting from the accretion of the primitive complex may also promote the formation of catalytic activity at its core. Due to specific internal components, small molecules bound to the surface of the complex can modify the intermolecular interactions within, thereby generating catalytic activity at the atomic level in the core through mechanisms such as orientation and electrostatic interactions. In addition, the active site may bind metal ions, which are crucial for the formation of the catalytic domain of the phosphodiester bond [26]. One or two metal ions may pre-exist in the catalytic core of the complex, as negatively charged RNA molecules usually bind many metal ions internally during folding [27]. Whether it is a polymerase or a ribozyme, their functions generally rely on the participation of these key metal ions [28].

Based on the characteristics of modern polymerases, it is speculated that the formation of phosphodiester bonds in the SCE assembly will lead to a conformational change in the entire complex, causing the base-paired nucleotides and the template RNA to move relative to the replicase. This conformational change and translocation may also result from further accretion-allosteric effects. A nucleotide moves out of the enzyme’s active site, leaving a vacant site that accommodates (selects) a new correctly paired nucleotide, triggering phosphodiester bond synthesis again. This cycle forms the basic replication function of the primitive RNA replicase. The interaction and reaction of the primitive replicase with its substrate resemble those of modern nucleic acid polymerases, and the replicase originates from a special self-assembled entity rather than being a product of selection. This special nature will be further explained later.

It has long been considered challenging to find a sufficiently long RNA capable of high-fidelity replication to replicate its complementary strand template. The threshold length of non-enzymatic RNA sequences required for replication activity is unclear, as almost nothing is known about this primitive replicase ribozyme. However, the accretion evolution model of stable complexes changes the rules, eliminating the need for a long RNA. Instead, multiple short RNAs of a certain length can self-assemble through accretion to form an enzyme-substrate complex, eventually evolving into an RNA replicase. Non-enzymatic RNA polymerization is rarely thought to exceed 20 nucleotides, although under specific conditions, spontaneous RNA formation may reach about 55 nucleotides [29]. For this reason, it has been proposed that RNA replication could arise from the joint action of many shorter strands [8,30], without the need for a single-strand general polymerase. Wu and Higgs [31] suggest that mathematical models of RNA polymerization chemical reaction systems indicate that short-strand complexes can form catalysts. The formation of such short-strand catalysts can be achieved through self-assembly in non-biological random polymerization systems, indicating a high likelihood of replicating complexes evolving from autocatalytic or mutually catalytic networks. Moreover, Carlos Briones and colleagues proposed a random polymerization model based on short RNA oligomers [32], which can evolve modularly through hairpin ligase-mediated reactions. In this process, hairpin regions in short RNA molecules connect two RNA units non-covalently or covalently through ligase-catalyzed reactions, forming longer RNA molecules. This may lead to the emergence of ribozymes with template-dependent RNA polymerase activity [32]. This model emphasizes the covalent or non-covalent connection of modular short RNAs by hairpin ligases, which may occur in parallel with the accretion evolution of replicase complexes. Clearly, RNA replicase activity is formed based on atomic interactions (specific combinations) resulting from the accretion of small RNAs, rather than relying on RNA genome-encoded mutations.

The accretion model of SCE is similar to the amphiphilic molecular assembly of GARD. Therefore, the GARD model can be utilized to compare and illustrate the RNA replicase resulting from the accretion in SCE. The composomes in the GARD form describe similar assemblies that repeatedly assemble in almost exactly the same way. Only when there is high affinity or minimized free energy between the components of GARD can the catalytic action between them be repeatable and selective. In other words, highly catalytic GARD assemblies will form repeatable composomes [12], and the mutual catalytic action between the components of the assembly in the GARD form is also considered to be a thermodynamically dominated binding [33]. It is generally considered that the strength of GARD lies in its ability to introduce supramolecular structures that can both replicate and evolve, while being simple enough to be simulated through detailed molecular dynamics [34]. In particular, the developers of GARD have realized that template replication is a special case. As a component of the assembly, the template promotes the integration of correct nucleotides and inhibits the incorporation of incorrect nucleotides [35]. Therefore, GARD considers the replication process as part of the metabolic network and does not need to separate it from other kinetic processes of the cell or protocell. In other words, replication is a special form of composome or enzyme assembly (Figure 3).

Additionally, Alexei Sharov argues that replication can be seen as a special case of autocatalysis applied to heteropolymers [36]. In this case, autocatalysis can be understood as the action of a set of reactions or heterogeneous assemblies. Each monomer is sequentially added to the newly constructed polymer according to a unified set of simple rules. Thus, autocatalysis can build template-based digital replication. Digital replication is considered universal because its replication rules apply to polymers of various lengths and any monomer sequence within the allowed range. However, the regularity of replication is determined by the internal components of the assembly. For example, the components of enzymes replicating the lagging and leading DNA strands differ. Hunding et al. [37] suggest that macromolecular aggregates (composomes) can have specific molecular compositions, such as lipids, oligonucleotides, and small peptides. The presence of chemical interfaces within complex composomes can create linear templates, based on which self-replicating molecules (e.g., RNA) can be synthesized, allowing the evolution of information replication through molecular templates. Hunding et al. also proposed mathematical and experimental tests based on this.

Clearly, the RNA replicase introduced by the SCE model is a special assembly that does not require selection. Is it possible for an assembly with potential replication capability to exist from the outset? First, this possibility cannot be denied or falsified. Among countless assemblies, such a combination is possible, especially in localized environments rich in nucleotide monomers and oligonucleotides. In fact, the possibility of prebiotic spontaneous generation relies on specialness, and the hypothesis of spontaneously forming replicating RNA is analogous to the spontaneous generation of catalytic networks based on prebiotic molecules. Kauffman’s (the CAS) autocatalytic set theory emphasizes that when molecular diversity reaches a threshold, catalytic closure networks inevitably emerge. In specific environments, an RNA replicase complex may also appear.

Second, this is a significant epistemological issue: is life special or universal? The conclusion is not so obvious, as the popular natural selection theory of biological evolution cannot be directly applied to the origin of life. Life may indeed be special, its origin accidental, and this applies not only to the first RNA replicase but also to the ribosome. However, the origin of life is not entirely random, as the structure of life’s origin (the first RNA replicase) “carries” a certain mechanism—intrinsic selectivity [17,19]—ensuring that the origin of life is almost certain. In other words, intrinsic selection mechanisms are a special form of matter, and biological substances like replicases and ribosomes follow this mechanism. This epistemology is not trivial; it influences researchers’ guiding principles and directs their research focus.

Third, from a philosophical perspective, the first structure of life cannot be a product of selection. If one insists on attributing a selective role to the origin of life, it can only be physical laws. As Eigen suggested, prebiotic reactions rely on random collisions and thermodynamic drives rather than adaptive selection [38]. Matter organizes into ordered structures through physicochemical reactions, likely driven by thermodynamics, or the earliest entities in the origin of life scenario are spontaneously formed assemblies, including networks of simple organic molecules [39]. It is believed that autocatalytic sets may organize into biological order, but this also requires explaining the challenging evolution of biopolymers and assemblies [40]. Physicochemical rules drive the formation of countless assemblies, one of which, with a special assembly, becomes the nucleating seed of life’s origin. Due to this randomness and scarcity, life on Earth or other celestial bodies (if any) may originate from a special assembly.

Fourth, replication systems require nucleotide base pairing with templates, and the simplest hypothesis is that this structure is included in the initial complex. Today, the bases found in life systems and their corresponding free nucleosides/nucleotides do not self-assemble through Watson–Crick hydrogen bonds in aqueous solvents. This issue is known as the “base pairing paradox” [41]. Therefore, the prebiotic process forming the first replicating complex may be limited to supramolecular assemblies capable of including nucleotide base pairing, ensuring that only molecules with pairing and stacking functions are incorporated into the complex as basic units [42]. Moreover, this pre-organized structure may easily synthesize phosphodiester bonds, as the compressive effects resulting from accretion may play a significant role when these groups are in the appropriate positions. Thus, this special structural aggregate provides the potential for replication.

## 3. Polymer and Metabolic Expansion of GARD

The GARD model can be extended mainly in two aspects: the extension from lipid assemblies to macromolecular assemblies and the formation of the general mechanism of the enzymatic catalytic activity of the assemblies, which are permitted by the GARD rules. The GARD theory models amphiphilic molecular lipid assemblies (such as micelles or bilayers) and proposes the concept of the “Lipid World”. The basic processes of GARD dynamics occur within the lipid phase, which includes the lipid bilayers of vesicles as GARD, as well as the GARD entities encapsulated in vesicles. Lipids are generally considered to be chemically inert, non-catalytic compounds whose main function is to form membranes. Although a membrane compartment is indeed required for the origin of life [43], GARD is not limited to this. In the scenario of the Lipid World, lipids and other amphiphilic molecules are actually considered to have significant catalytic functions [44]. For example, heterogeneous micelles are similar to globular proteins, having similar sizes, hydrophobic interiors and hydrophilic functional surfaces, and they also possess catalytic capabilities [44]. The combinatorial proximity of headgroups may generate catalytic centres on the surface, making the surface of lipid assemblies an alternative to the mineral surface, but with higher flexibility and diversity [45]. Lancet et al. introduced the term “lipozyme” to refer to lipid molecules with the ability to enhance reaction rates. Almost all cases of GARD fall within the category of non-enzymatic catalysis (or enzyme mimetic catalysis) [14]. However, covalent catalysis is necessary for the transition from chemical evolution to biological evolution, which may be mainly achieved through polymers of nucleotides or peptides, as shown by modern biology.

On the other hand, although GARD can embody two widely accepted cornerstones of the origin of life, namely compartmentalization and information storage/replication, the third cornerstone, metabolism, is currently only partially modeled in GARD through non-covalent reaction networks [9]. Although non-covalent reactions are real participants in modern metabolism, typical examples include ligand binding, protein folding, conformational transitions, and solute transmembrane transport. However, it is obvious that for a full analogy with modern metabolism, GARD must be compatible with covalent reactions [9]. Although there are many cases of lipid catalysis, such as the introduction of prebiotic non-enzymatic catalysts, including cofactors (coenzymes), which are hypothesized to be the early precursors of modern enzymes. An important criticism is that as long as GARD is limited to non-covalent chemistry, its ability to demonstrate full evolutionary characteristics is limited [46]. Therefore, GARD needs to be extended with polymers to enzymes capable of characterizing covalent chemistry or stereochemistry, evolving from mainly describing lipid assemblies to assemblies compatible with nucleotides and peptides. Moreover, the GARD theory clearly states that amphiphilic molecules are not limited to lipids [18,44], thus allowing the extension of GARD to nucleotide or peptide assemblies. According to the SCE model, these GARD assemblies can form enzyme-substrate complexes and supramolecular structures of functional assemblies, and this is necessary for the GARD model to cross from characterizing chemical evolution to biological metabolic evolution. As the GARD developers themselves said, the origin of life necessarily involves an unlikely transition from chaotic chemical reactions to self-replicating supramolecular structures [14]. Currently, the synthesis of oligomers or macromolecules from monomers has been considered in the GARD model, but there is a lack of modeling on how macromolecular polymers generate enzyme activity through mutual catalysis/binding.

To achieve functions closer to modern metabolism, GARD attempts to gradually expand to the Polymer GARD (P-GARD), also termed Metabolic GARD (M-GARD) [9,47], introducing covalent reactions and polymer formation mechanisms. In P-GARD, dimers and higher polymers can be formed. These polymers can replace some monomers, undertake catalytic roles in the network, and may exhibit higher catalytic efficiency. The formation of biological macromolecules (such as RNA) may depend on controlled oligomerization based on composomes [9,12]. In the M-GARD framework, it is considered that nucleotide lipids can synthesize oligonucleotides through continuous covalent modifications [9], or they can be synthesized by other abiotic means, such as the action of clay surfaces and alternating wet-dry environments. M-GARD further expands molecular diversity by endogenously catalytically synthesizing new compounds. These compounds participate in GARD dynamics and pass on this catalytic ability from generation to generation, partially solving the problem of catalyst transfer in metabolic replication. Although metabolic networks can provide necessary compounds for biopolymers, the emergence of the RNA system is the key to realizing genetic evolution [48]. Regarding this, Orgel [5] believes that it must be assumed that a library of RNA chains with different sequences was spontaneously (autocatalytically) formed on the primitive Earth, and this family of sequences includes catalysts capable of supporting RNA self-replication.

Biological systems are capable of autocatalysis and self-organization, which means they can metabolize, reproduce, and produce more of their own components, and it is biological polymers that enable organisms to achieve this. Therefore, a very important goal is to understand how autocatalytic and self-replicating biological polymer systems emerged from non-living chemical systems. Although the theory of collective autocatalysis is also considered to be capable of self-replication, collective autocatalytic sets usually do not emphasize the catalytic role of polymers. In this case, the rationality of autocatalytic cycles has been questioned by people [49]. Not only for metabolism, but cells also use biological polymers to store genetic information. The transformation from small-molecule mutual catalytic networks (dependent on compositional information) to genetic polymers (dependent on sequence information) is not clear. Due to the existence of genetics, organisms can produce the catalysts required for metabolism and also enable the genetic polymers themselves to replicate. Conversely, the metabolic reaction network ensures the expression and regulation of genetics. It is precisely because biological polymers make it possible to coordinate genetics, metabolism, and evolution that life can be self-sustaining. Therefore, it is believed that the emergence of autocatalytic biological polymer systems is one of the key steps in the origin of life.

Undoubtedly, the presence of biological polymer catalysts is an important characteristic distinguishing biological systems from non-biological chemical systems [50]. An unclear issue in the GARD model is stereochemistry, that is, the lack of an extension of the general principles of enzyme kinetics. It is precisely at this point that GARD can be extended through the accretion evolution model of SCE, and GARD will thus be able to characterize enzyme kinetics. The key to connecting the two models is the combined action of mutually catalytic self-assembly and accretion, and enzyme complexes can be understood from this perspective. In fact, GARD’s description of the mutual catalysis between components is broader and more general. A special case of it is the formation of stereochemically enzyme-catalyzed complexes, which needs to be understood at the level of nucleotide and peptide assemblies. What makes RNA special is that it can both carry genetic information and have the ability to catalyze reactions. Its structure is usually constructed from secondary structures formed by a large number of base-pairings. Some short or slightly longer RNA sequences may fold or pair with each other to form smaller assemblies, which further form larger structures. As the SCE model shows, this accretion can generate catalytic activity. According to the GARD model, an RNA accretion complex is a mutually catalytic or interacting set without considering their specific sequences. When binding specific substrates, the accretion complex can generate stereochemical catalytic activity. This may be the basic way for the transformation of GARD into covalent catalytic assemblies.

As mentioned above, the first replicase was formed through the accretion of multiple heterogeneous molecules with various intermolecular interactions, and it can be regarded as a mutually catalytic GARD assembly. The GARD model supports the analogy between equilibrium affinities and catalytic rate enhancements [9,12,51]. That is to say, the binding affinities between components in GARD can be analogized to catalysis. Similarly, other enzymes (such as helicase ribozymes) can also evolve in a similar way. Specifically, multiple short RNAs stably bind to a specific RNA structure through accretion, thereby forming the catalytic activity to unfold its folding. Moreover, all modern enzymes can be analyzed from this perspective. That is, we can view the long RNA/peptide chain structures with multiple domains as a form in which multiple sequences are covalently linked and self-folded. The interactions during the self-folding process can be described as non-covalent and complementary high-affinity binding. And stereochemically, the binding of the folded long sequence to the substrate is equivalent to the accretion binding of multiple shorter sequences to the substrate.

The template-dependent nucleotide polymerization catalyzed by highly specific RNA assemblies will not be limited to using itself and its complement as templates, and other RNA molecules in the environment will also be replicated. This replication will generate extensive diversity, and some replicated RNAs may have useful enzymatic activities, such as specific RNA-processing enzymes like RNase P. Alternatively, certain RNA molecules may be able to bind amino acids and part of the RNA template, thus acting as primitive transfer RNAs [52], which may provide materials for ribosome evolution. In this way, protocells can establish a system composed of many ribozymes, all of which are replicated by the same polymerase. In the autocatalytic system, the relationship between the sequence specificity and generality of replicators is an important unresolved issue [31], which is considered to be a difficult problem to solve. As mentioned above, the RNA replicase produced by the accretion model is a general polymerase. And judging from modern protein RNA replicases, this RNA replicase does not require a primer and may not require a specific polynucleotide initiation sequence either. Therefore, the RNA replicase ribozyme with high-affinity interactions seems to be all-powerful. Of course, since the RNA template strand is often in a folded state, it is not easy to replicate its full length. Therefore, the primitive RNA replication system may need the cooperation of helicases. As mentioned earlier, we can speculate on the evolution of RNA helicase ribozymes based on the SCE model. It can be seen that the extended GARD model can be a general mechanism for enzyme formation, which provides a general solution framework for various individual evolutionary problems.

## 4. Repeated Assembly of Replicases

GARD, which has a research history of over 20 years, is a mutually catalytic network. It is described by kinetic equations and suitable for computer simulation and is composed of self-assembling amphiphilic molecules. GARD aggregates maintain a dynamic non-equilibrium through molecular exchange. Its steady-state growth rate is determined by the current composition and regulated by the matrix β for kinetics [9,12,53] (see Figure 1 and Figure 2). This is both the result and prerequisite of compositional inheritance, and combined with random division, it can achieve compositional self-replication. Only composomes with specific molecular compositions have this characteristic. When modern cell lipid bilayers divide, their compositions basically remain unchanged, which is the best illustration of the possibility of GARD’s steady-state growth. Lipid vesicles and protocells are regarded as composomes if self-reproduce and thus their assembly can be repeated. Of course, the maintenance of steady-state growth in modern cell membranes is not entirely determined by the internal catalytic kinetics of the membrane. It requires the synergistic action of other sub-networks (systems) in the cavity. This synergistic action can be characterized (through expansion) by the GARD model, as pointed out in a reference [54].

So, how does the first replicase in the SCE model replicate itself? According to the GARD model, the replicase is a composome because it is an assembly that can self-repeat and keep its composition basically unchanged. According to the SCE model, the self-assembled stable complex has minimized energy, including the interactions between components. It is this feature that ensures the “replication” of the first replicase, or rather, the self-assembly of a new replicase. This is not the self-replication of the full-length RNA sequence of the replicase, but the spontaneous assembly of the replicase components to form an almost identical replicase. Specifically, since the first replicase can replicate any single-stranded RNA substrate, and the pre-biochemical environment is stable (this assumption is necessary for the origin of life), all kinds of components of the replicase can be obtained again (or multiple times), and the reassembly of these components occurs in sequence. The key to this process is that the SCE assumes that the binding between each component of the replicase has minimized energy, so there is high affinity and specific recognition between the components.

Repeated assembly has relatively strict requirements for the component sequences or structures. For example, the first RNA component that constitutes the RNA replicase complex can be repeatedly obtained, and it has sites for binding two nucleotides and a single-stranded template. After the formation of this initial complex, the other accreted small RNA components also need to remain basically unchanged in terms of sequence or structure, which is possible under stable pre-biochemical conditions. First, for relatively short RNAs, in the pre-biochemical environment, their sequence space is limited and highly repeatable [55]. Since chemical evolution occurs in a much smaller sequence space, all possible variants may exist from the very beginning. Moreover, chemical evolution is repeatable, while biological evolution is not. Second, studies on RNA folding support the repeated assembly of RNA replicases. In a certain environment, RNA usually folds into a structure with the lowest energy state, i.e., the native state. However, sometimes RNA may fold into an incompletely optimized (non-lowest energy state) structure, which is the non-native state, but they can also refold into the correct conformation (native state) after a certain period of time [56]. Considering only the RNA’s own sequence and the binding ligands, RNA folding is highly repeatable because the native folding is the state of lowest free energy. Third, RNA structures have certain commonalities. That is, a small number of base mutations in an RNA sequence of a certain length usually do not change its structure. Therefore, this allows the RNA replicase to use RNAs with slightly altered sequences during the recombination process. Ribozymes mainly function in a structural manner. If the structure remains intact, ribozyme activity can be maintained [57,58], and it can even tolerate minor structural mutations [59]. In the replicase complex, the RNA components may mainly interact with each other in structural forms such as stem-loops and hairpins. Searching for appropriate structures is more meaningful than searching for sequences because for a given sequence length, many different sequences fold into the same structure [59,60]. Fourth, some RNA structures are more common than others. For shorter sequences (about 30 nucleotides in length), more than 90% of the sequences fold into common structures [61]. Although there are many other structures outside the common ones, they are only represented by a small number of sequences. These rare structures are difficult to find because they only exist in certain corners of the sequence space rather than being ubiquitous common structures. Therefore, the possibility of these rare structures being incorporated into the replicase complex is relatively small. Moreover, through evolution, common structures can be easily reached from any starting point in the sequence space [62]. Therefore, finding a specific structure through mutation and selection is much simpler than expected. Even if catalytic activity is associated with rare RNA structures, it is unlikely to be missed by the evolutionary process [60].

The process of repeated assembly of RNA replicase may undergo adaptive evolution. During the repeated assembly process, incorrect components cannot stably bind to the replicase complex, and this is considered to indicate that the SCE functional complex has self-selectivity (intrinsic selection). Similarly, the composome in the GARD model is also regarded as having internal molecular selection [9], as the composome tends to retain its existing components. It is believed that molecules with altered folding usually do not integrate into the aggregate [63]. Moreover, at any given position in a stable aggregate, only one monomer (component) is active (correct); all other monomers (components) at that position are inactive [49]. Therefore, it can be speculated that when there is a lack of a correct component at a vacant site in the assembly, it will wait and select a correctly folded component, or accept a slightly altered component through minor changes in adjacent components. This selectivity is the result of following physicochemical principles rather than biological selection, and it can play an important role in the chemical evolution stage.

In the absence of a sequence-based genetic system, the repeated assembly of RNA replicase may be slow. As mentioned above, the primitive replicase complex may consist of multiple RNAs, which requires waiting for the correct components to bind to the assembly. Moreover, some small RNAs that are misfolded and cannot bind stably may need to be replaced, or correctly folded molecules may be covalently linked into longer chains by specific hairpin ribozymes or ligase ribozymes. Therefore, the replicase components may gradually become longer and be selected for assembly stability, and these ligated RNAs are ultimately maintained through replication by the replicase. This may be the reason why some functional RNA molecules ultimately have longer RNA sequences. Meanwhile, this repeated assembly indicates that the expansion of the primitive replicase is not exponential. It simply repeatedly constructs some basically similar but functionally identical RNA replicases in a stable pre-biochemical environment.

## 5. From Compositional Information to Sequence Information

Compositional inheritance is an important concept in GARD [33,64]. The GARD model regards compositional information as a genetic carrier equivalent to sequence information, similar to the function of sequence information in sequential biopolymers in the RNA and protein world. In traditional genetics, genetic information is transmitted through the sequence information of DNA or RNA, that is, a specific nucleotide sequence encodes the protein-controlled genetic characteristics of an organism. In compositional inheritance, genetic information is transmitted through molecular composition, that is, the proportions and combinations of specific molecular types determine the functional characteristics of the assembly. Compositional information assumes that all assemblies (with a total number of molecules N) composed of N_G_ molecule types have the same amount of compositional information, but a specific composition (such as a composome capable of efficient replication) confers functional differences, similar to how a genome encodes biological functions. Through theoretical calculations, when the number of molecular types (N_G_) is low, the coding efficiency of sequence information is higher; but when N_G_ is high (which may be relevant to the origin of life), the coding efficiencies of the two types of information gradually converge, as described below. Importantly, compositional information is daily used in what is often called epigenetics, namely proteome, transcriptome and metabolome of a living cell. Compositional information and sequence information are not mutually exclusive. For example, a compositional assembly can contain sequence molecules (such as peptide chains or oligonucleotides), and the mRNA population in cells can also be considered within the scope of compositional information analysis (such as transcriptome analyses) [65]. Therefore, the molecular composition of the composome is analogous to a “genome” and composome is derived from the “compositional genome”, which determines the replication ability and phenotypic characteristics of the assembly through specific molecular ratios. The compositional genome realizes its functions through a dynamic interaction network (such as mutual catalysis) to form a rudimentary phenotype, for example, promoting self-replication or maintaining structural stability.

Some argue that, despite the merits of the GARD model, it has not yet provided a sufficiently universal framework to help us understand how chemical evolution transitions from compositional information mostly non-genetic information to genetic to dual composition/sequential information role [66]. As mentioned in Section 3, in the realms of Metabolic GARD and Polymer GARD [9,47], many of the molecules in a composome may actually have oligomers of any kind, including peptides and oligonucleotides. This suggests that GARD composomes as well as SCE assemblies may be referred to by both composition and sequence. This means that there is no jump from composition to sequence, and that the evolutionary process that took place must have involved a gradual change in favor of sequence over composition. While GARD composomes strongly depend on mutual catalysis among small molecules, including oligomers (often headgroups of lipids), at a later point in evolution, with molecules that constitute long polymers, the catalysts are termed “enzymes”, which strongly depends on their sequence.

The metabolic GARD opens a path of gradual transition between compositional information to sequential information. It is important to stress that, in the present context, information is simply what is copied when a molecular assembly is self-reproduced or self-replicated. While information transfer from RNA to protein is mediated by a genetic code, there is no expectation that any translation mechanism would transition the composition of a micelle or a vesicle to the sequence of a polymer. Instead, what happens is that at the surface of lipid assemblies, new entities begin to appear, such as oligomers, or even polymers (Figures 5 and 6). This marks the entry of covalent metabolism that may allow catalyzed oligomerization (see Section 3). When this happens linearly, and with a limited repertoire of monomers, it is possible to use the term “sequence”. The only phenomenon revealed in this process is that the monomers are those that are within the vesicular lumen and in the membrane, or in the environment. In addition, the catalytic events involved are local. But clearly the transfer from lipid assemblies to the emergence of oligomers is a critical step for enhanced complexity, mostly expressed by gradual emergence of supramolecular entities and kinetic networks as well as polymers. We note that the magnitude of N_G_, the size of the count of small organic molecule types (monomers) in the prebiotic environment is largely irrelevant to how complexity in early life increases.

**Figure 4:**
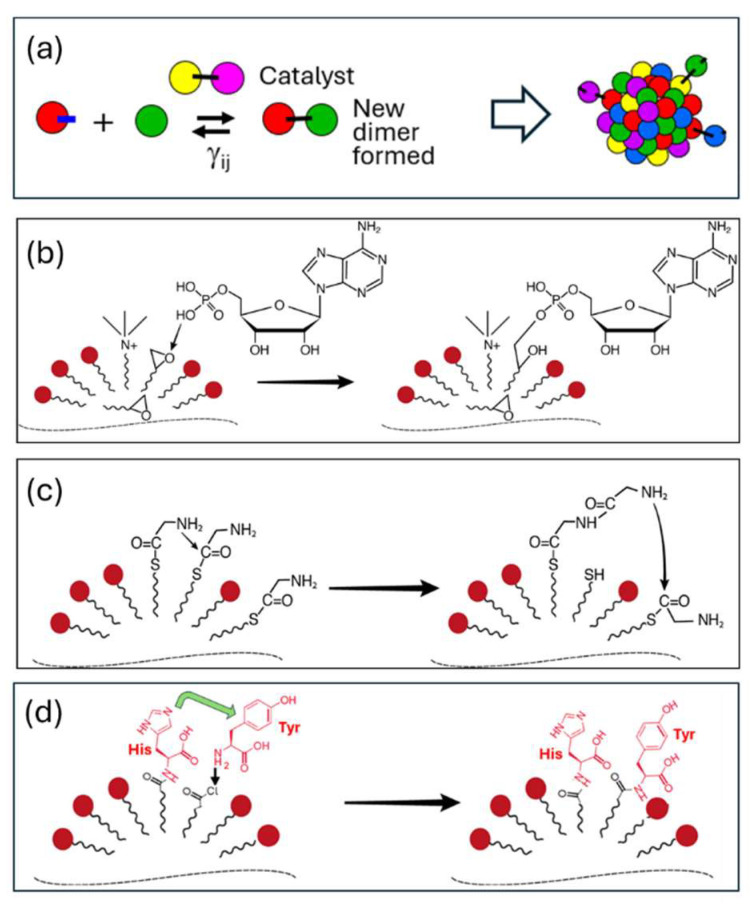
(**a**) the basic GARD model, which involves a mutual catalysis network, focuses on non-covalent reactions, typically joining and leaving the of monomers in lipid layers. However, there is strong evidence that GARD may also apply to covalent reactions (“metabolic GARD”), whereby two monomers form a dimer [9,18,47,67]. The capacity to reach a reproducing micellar composome, i.e., preserving catalyzed covalent binding reactions, is shown by computer simulations (P-GARD, [9]), where colors indicate different lipid types. Three examples of such specific reactions that are observed experimentally (**b**–**d**) are reactions on micelle lipid surfaces. These are: (**b**) covalent binding of a nucleo-lipid formation), (**c**) The forming of a lipid-bound tripeptide (with a potential longer extension) of glycine amino acids, covalently bound to the lipid surface, (both from [67]. Last, in (**d**), covalent bonding of a tyrosine amino acid to activated lipid tail, forming a new lipid, a reaction catalyzed by a lipid with a single histidine lipid head group [68].

**Figure 5:**
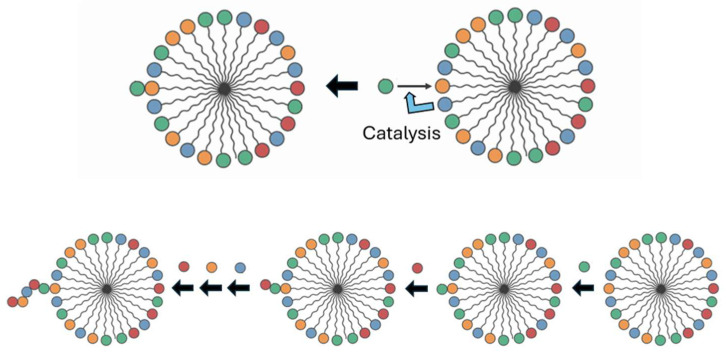
Based on the information in Figure 2, catalyzed covalent reactions on the surface of a micelle, an entity defined by its compositional information, may grow oligonucleotides, oligopeptides and likely also other oligomers that are defined by their sequence information. Importantly, the successive monomers that add to the growing oligomer may each be catalyzed by another headgroup, as lipids phases are often fluidic (the color of headgroups and oligomer members indicate different chemical structures). This situation is, to some degree, analogous to bacterial non-ribosomal peptide synthesis [69].

**Figure 6:**
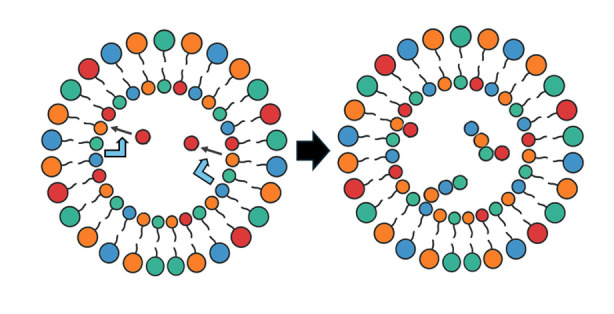
Similarly to those of micelles, covalent modifications have been reported for cases that modify vesicular membrane bilayers. Reactions occurring in vesicular membranes, there are lipid covalent modifications [70]. This supports the probability of sequential modifications that generate oligomers. Particularly relevant is the fact that catalyzed reactions are, as always, reversible (**left**). Thus, if an oligomer is growing in the lumen, on the inner bilayer, oligomers may grow, and then a reversible reaction releases the oligomer into the lumen (**right**) (the color of headgroups and oligomer members indicate different chemical structures). This generates a probability that some oligomers, e.g., oligonucleotides, could gain functions.

Figure 4a introduces the concept that lipids may interact covalently and under the catalysis of neighbor molecules, e.g., forming dimers between headgroups, or between lipid headgroup and other small molecules. Some experimental results are shown in the rest of Figure 4, where on the surface of micelles there are reactions that lipids can be linked covalently to interesting other molecules. In Figure 4b a lipid with headgroup of a nucleotide is synthesized, in Figure 4c there is a synthesis of an oligopeptide (oligo glycine), and in Figure 4d a lipoamino-acid with a tyrosine headgroup, with a neighbor lipoamino-acid of histidine. Based on these experiments, we generalize some capacities in Figure 5. Here, a consequent covalent threading of entities such as nucleotides or amino acids can form oligos that have sequences. In Figure 6 we portray the idea that if such reactions happen on the inner leaflet of a membrane of a vesicle, such oligomer chains can become residents of a protocell with certain functions, such as short early RNA or protein, with certain functions that depend on sequence. Thus, in the scene of GARD-behaving reproducing micelles, the composition–sequence enigma may begin to be solved.

## 6. Attractor Properties and Selectivity of Replication Systems

**Figure 7:**
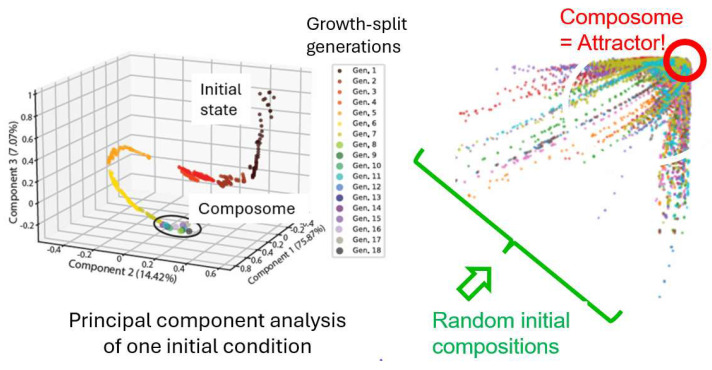
**(Left**): Beginning with random composition, the micelle grows and fissions 18 generations, and then gradually approaches the composome. (**Right**): The diagram shows that many such random initiations end up in a narrow target, at which the micelle undergoes self-reproduction, i.e., reaches by definition a composome. This means that the composome is a dynamic attractor!

In the GARD simulation, an amphiphilic molecular assembly with random composition forms spontaneously, followed by growth-division dynamics driven by the entry and exit of environmental monomers. As the simulation continues, one of the division products is retained to maximize the conservation of reaction substances [71]. In some cases, the system reaches a privileged compositional state, namely the composome [12], in which a high degree of compositional conservation is observed. The compositional invariance observed in the growth and division cycles during the simulation meets the criteria for assembly replication [9]. Composome reproduction has been recently shown by GARD simulations to own dynamic attractor properties [14] (See Figure 7).

Composome replication is considered to be due to its attractor properties. This dynamic attractor refers to the stable state that the system tends towards during the evolutionary process, and many initial states will eventually converge to this state. In the context of the origin of life, the dynamic attractor can be understood as a self-maintaining chemical entity that can spontaneously emerge from a randomly chaotic environment [14]. That is to say, there is a close relationship between the dynamic attractor and the composome. The dynamic attractor defines the final stable state of the system’s evolution, while the composome is the specific chemical composition in this state. Therefore, for the prebiotic catalytic network model, the system will evolve from diverse initial compositions and converge to the composome, which reflects the guiding role of the dynamic attractor in the system’s evolution.

The convergent state of attractors not only guides the system evolution of prebiotic catalytic networks but also plays a guiding role in the evolution of biological systems. A key aspect of today’s life is the complex network of interacting components that exhibit attractor behavior [14]. Meanwhile, the metastable dynamic attractor states of life maintain non-equilibrium with the environment and convert food and energy into their key components [72]. Complex biological networks often display multiple high-dimensional dynamic attractors. For example, co-existing attractors have been observed in the metabolic networks of self-organized multi-enzyme complexes [73]. Attractors also exist in other systems, such as in the fields of signaling protein networks [74], cell differentiation [75], and transcriptome regulation [76]. The authors suggest that through coordinated catalytic interactions, metabolic networks may transition between different quasi-stable attractor states in response to external and internal stimuli. Numerical solutions of GARD’s differential equations indicate that GARD compositional reproducers have been found to transform into each other through compositional mutation dynamics [71]. According to the current interpretation, this means that GARD networks typically have multiple attractors. Current GARD simulations also show behavior similar to that of gene regulatory network attractors [77], where changing the concentration of key environmental chemical components leads to a shift from one attractor to another.

It is reasonable to speculate that all biological systems or subnetworks are attractors, as changes in the behavior of living cells can be interpreted as switches between attractor basins [74,78,79,80]. So, how are attractors formed? Given appropriate diversity and catalytic distribution, dynamic attractors can be formed in at least two ways: supramolecular entities with attractor properties that are highly likely to regenerate in the prebiotic environment [14], which may be related to the overall energy minimization; or dynamic attractors formed by specific autocatalytic networks [72].

Similarly to composomes, SCE assemblies are also a type of attractor. According to the SCE theory, all biological functions originate from stable complexes, and they are all dynamic attractors [19]. The definition of SCE assemblies is based on the minimization of energy through the interactions among all their components (thermodynamic encoding). Meanwhile, it also encodes information about the transformation from thermodynamic control to kinetic control (kinetic encoding). Therefore, it is essentially a dissipative nonequilibrium system. Specifically, when an SCE assembly approaches thermodynamic equilibrium (driven by thermodynamic encoding), the kinetic control reactions encoded within it (such as transition state stabilization) will disrupt this equilibrium and trigger catalytic reactions. Subsequently, in a new cycle where the catalytic products dissociate and recombine with the substrates, the SCE assembly tends to return to a thermodynamically stable state. The transformation of kinetic encoding must occur on the basis of the stable complexes of thermodynamic encoding. This internal encoding determines the spontaneous occurrence of the functional cycle (nonequilibrium steady state). Non-enzymatic functional structures can achieve non-equilibrium steady-state cycles through allosteric effects. Therefore, this process forms an attractor with the thermodynamic stability of the SCE assembly as the convergent state [19].

The GARD model has clear thermodynamic and kinetic simulations [81], although it does not emphasize the transformation from thermodynamic to kinetic encoding in the component information of composomes. Additionally, as a pre-RNA life model, GARD has been observed to exhibit thermodynamic and kinetic characteristics. These characteristics include an attractor-like transition from random assembly to self-organized complexes, which involves a negative entropy change. These properties, combined with the system’s constant state far from equilibrium, its exchange of matter and energy with the environment, and its ability to amplify small fluctuations, have established GARD composomes as a dissipative system [9]. Therefore, this process reveals a key mechanism: when an assembly can stably carry out replication or regeneration reactions, it is an attractor and maintains a dissipative nonequilibrium steady state through the transformation from thermodynamic control to kinetic control. This is also the fundamental way for biological systems or networks to transform from thermodynamic control to kinetic control.

An obvious trend has been observed: only networks with a high tendency for mutual catalysis can increase the diversity of composomes (quasi-stable compositions showing high replication fidelity). Importantly, this mainly occurs when there is a relatively high proportion of mutual catalysis in the GARD network [13]. Moreover, experiments have shown that the rate of reaching the regeneration attractor increases as the catalytic ability in the network strengthens [14]. The results highlight the potential important role of strong mutual catalysis in the emergence of early life-like systems. When such strong catalytic action is understood as affinity, it leads to the formation of SCE model assemblies with high affinity.

The typical characteristics of attractors explain the tendency of the replication properties of GARD assemblies and other chemical networks to emerge [40,82], and this is also a tendency towards self-maintenance and self-stabilization. Taking a specific type of living cell as an example, an attractor is characterized by the ability to maintain a repetitive pattern [83]. According to the widely accepted definition of an attractor [84], in biological systems, the first characteristic of an attractor is being in a stable steady state, towards which many initial states ultimately tend to converge [75,85]. The second characteristic of attractor dynamics is resilience to disturbances, that is, the ability to quickly return to the steady state after being disturbed by internal or external factors [86,87]. This property is demonstrated in the study of biological attractors, where a steady-state cellular system is perturbed by a controlled stimulus and then returns to the attractor state. Similar phenomena have been observed in multiple catalytic networks, showing different but significant levels of resistance.

The stability and anti-interference properties of attractors are the basis for the system to form order and undergo evolutionary selection, which is also the core inference of SCE assemblies. Similarly, the GARD model proposes that the driving force for the origin of life is closely related to the formation of attractors [14], which is a highly forward-looking view. The early stages of the origin of life involve the emergence of ordered and complex structures from relatively disordered chemical reactions. This evolutionary process requires a special driving force, which may manifest as a process of random formation and selection, or a mechanism related to autocatalytic networks that can promote the formation of attractors. By emphasizing the central role of attractors in the origin of life, the SCE and GARD models provide a new theoretical framework for understanding the transition from chemical chaos to biological order.

The above discussion can be imagined to be tested through artificial selection and experimental testing or demonstrated within a mathematical framework. For example, specific conditions or sequences can be added in artificial selection and experimental simulations to enable better formation of stable interactions and attractor properties among the components of the assembly. Moreover, targeted settings can be made for the configuration of the core part of the self-assembly to pursue specific chemical activities. It is worth noting that the core of the assembly must achieve tight binding. Only high affinity can induce kinetic transformation, or in other words, achieve stable and reproducible catalysis. In this way, the hypothesis that the reproducible assembly produces catalytic activity can be verified. This seems feasible, especially in this AI-assisted era.

**Figure 8:**
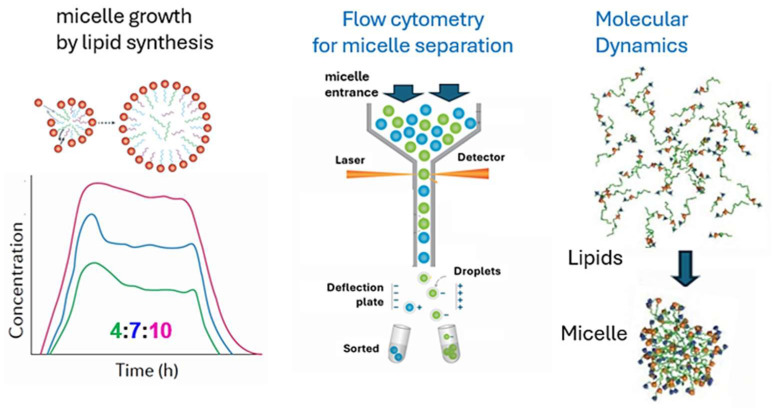
The set of experiments done for micelle growth are carried out in a flow chamber technology [86]. There are three types of lipids, defined by three different tail lengths, colored differently., that can get synthesized and accrete to micelles. This experimental path shows that as long as the chamber is stirred, there is a steady state that keeps the composition constantly at a ratio of 4:7:10, i.e., homeostasis. This technology is already successful and surely has a successful future. The second hopeful technology is flow cytometry that allows to sort micrometer size cells of different kinds. By measuring the population of each kind, one can tell which composition reproduces better than others, i.e., which generates a species. Applicability for micelles is upcoming, as the method is now developed for nanoscopic capacities [88]. The third futuristic technology is Molecular Dynamics, which we have already begun to use [34] for micelle formation. Experts predict that by the next decade, this computational approach will be able to simulate the dynamics of an entire bacterium, with its thousands of molecule types and their interactions [89]. This will easily allow to accurately follow the behavior of supramolecular entities which this paper deals with.

Finally, it is noteworthy to comment on testable predictions to transition the work from a theoretical discussion into a falsifiable framework for future experimental or simulation-based research. Figure 8 portrays three relevant technology fields in the scope of GARD, for sure also applicable to SCE. All three techniques that follow the fate and quantitation of supramolecular aggregates that may take part in the early steps of life’s origin.

## 7. Conclusions

The GARD model involves three fundamental concepts of the origin of life: replication, heredity (evolution), and metabolism, and explains these concepts through GARD lipid assemblies. Although compositional replication is not a mainstream concept in early-life research, it is ubiquitous in modern biology [89]. An important inference of the GARD model is the proposal of the concept of composomes, which are the dynamic states of compositional assemblies. They not only exhibit metabolism-like characteristics but also have the initial ability to store and transmit molecular information [9,12]. Moreover, the concept of composome not only reveals a mode of replication but also indicates the fundamental role of stable and reproducible structural entities in the origin and evolution of life. The GARD model suggests that these composomes bridge the gap between the two opposing hypotheses regarding the early origin of life: “metabolism first” and “replication first”. As described earlier in this paper, the extended polymer model of GARD can be formally equivalent to the enzyme assembly of the SCE model. Therefore, “metabolism first” and “replication first” are not mutually exclusive but complementary.

By introducing the SCE model, when GARD is extended to the assembly of oligonucleotides, it can integrate mutual catalytic networks and template replication within a single conceptual framework. Both the SCE and GARD models support the existence of compositional information and believe that to some extent, compositional information reveals the essence of biological heredity. Biological information is attached to structure and function, and in the early stage, compositional information was unified with structure and function, that is, the genotype and phenotype were the same. The evolvability of the primitive replication system is related to the attractor properties of mutual catalytic networks or self-assembling complexes, and evolution is a change under the constraint of attractor stability. That is to say, an attractor with overall stability has selective properties, and when disturbed, it selects changes that brings it back to the attracting trajectory. In this sense, compositional information can change. For example, the coordinated change of two related components does not affect the overall stability. Therefore, stability takes precedence over compositional invariance. Of course, attractor dynamics maintains the stability of compositional information, not the invariance of all components.

Methodologically, the SCE model can be compared with the typical GARD model. Firstly, like GARD, SCE assemblies can be mainly composed of amphiphilic molecules, such as short peptides and oligonucleotides, and in this paper, nucleotides are mainly considered as components of SCE assemblies. Second, the components of SCE assemblies are mainly bound through non-covalent complementarity, which can also be understood as a catalytic effect, just as set in GARD. One molecule can increase the incorporation rate of another molecule, such as through specific binding (high affinity); or it may accelerate the departure of a molecule. The allosteric or catalytic effect of molecule incorporation leads to the departure of components. Third, SCE assemblies are similar to GARD composomes. They are defined as complementary interactions and stable aggregates. Therefore, they can repeatedly self-assemble while keeping the basic components unchanged. Fourth, like GARD, SCE assemblies exclude sequence inheritance dependence and mainly function in a structural form. An important feature of the GARD model is that it ignores sequence information. The function and replication rate of the assembly only depend on the intermolecular interactions and the current composition. Fifth, the enzyme assemblies of SCE are attractors with reaction cycles and networks. They always tend to self-assemble (replicate) with the same components and have certain anti-interference ability and plasticity. This is consistent with the composome. Sixth, like GARD, self-catalytic network can be formed between SCE assemblies. This can be understood at a higher level. For example, the generation of each component can be achieved through multiple coordinated and associated SCE assemblies, and both the catalytic substrates and enzymes can be regenerated within the system. Seventh, to some extent, SCE assemblies can divide and proliferate. The process of an enzyme catalyzing the conversion of a substrate into a product can be regarded as asymmetric division. During this cyclic process of division and reassembly, adaptive evolution and compositional inheritance may occur. The division and reproduction of GARD entities can occur at the supramolecular level and the protocell level [67], and the reproduction of protocells may exist, but the role of natural selection through reproduction in the early evolution of the origin of life is controversial. Perhaps the internal selection recognized by both the GARD and SCE models is an important evolutionary factor in this case. Eighth, SCE assemblies are compositionally heritable. The first RNA replicase of life was constructed from dispersed information molecules. This compositional information can be transformed into DNA genetic information during the evolution of the RNA replicase itself, as shown in reference [17]. In summary, by comparing and integrating the well-studied metabolism-first GARD model with the replication-first SCE model, we propose a scheme for the evolution from oligonucleotide assemblies to replicases.

## Data Availability

No new data were created or analyzed in this study.

## Abbreviations

1. Accretion	The binding or interaction of molecules in a specific sequential order.
2. Attractor	An attractor is a stable state or set of states in a dynamical system toward which the system tends to evolve over time, regardless of its starting conditions within a certain region (the basin of attraction).
3. Assembly	Small molecules spontaneously assemble into stable aggregates in a specific manner.
4. Biological Evolution	An evolutionary mode based on biological forms not only exhibits a continuous transition with chemical evolution but is also not limited to natural selection.
5. Chemical Evolution	A series of abiotic chemical reactions on early Earth led to the formation of increasingly complex organic molecules, ultimately laying the foundation for the origin of life.
6. Collectively Autocatalytic Set (CAS)	It is a system of molecules where each molecule can be produced by at least one catalytic reaction within the set, making the entire network self-sustaining and capable of reproduction.
7. Compositional Information	The pattern of chemical composition within a system—such as the types and proportions of different molecules can itself store and transmit information.
8. Composome	A system or composite entity with specific components that can self-reproduce.
9. Graded Autocatalysis Replication Domain (GARD) Model	It is Lancet’s computational framework that simulates how collections of mutually catalytic molecules (e.g., lipid-like assemblies) can grow and fission, mutate, self-reproduce, exhibit rudimentary compositional inheritance, and selection without nucleic acids, offering a possible prebiotic pathway to early biological evolution.
10. Genetic Information	Linear instruction information encoded by nucleic acid sequences (such as DNA/RNA) and accurately transmitted through template replication.
11. Kinetic Coding	Following thermodynamic coding in the SCE model, transition-state stabilization and catalytic conversion that occur due to the internal specific RNA sequence configurations that lead to chemical transformation and/or allosteric changes.
12. Metabolism-first	It refers to the hypothesis that life originated from self-sustaining and evolving networks of chemical reactions (early metabolic cycles) before the emergence of genetic replication mechanisms like RNA.
13. Metabolic GARD (M-GARD)	The metabolic form of GARD. A version of the model in which not only entry and exit into assemblies occur, involving only non-covalent binding, and covalent synthesis also occurs.
14. Mutually Catalytic Network	A network system formed by intermolecular mutual catalytic interactions, serving as one of the models for the transition from prebiotic chemical systems to living systems.
15. Oligonucleotide	A type of short-chain nucleic acid polymer, (which can be DNA or RNA), typically composed of 5-50 nucleotides.
16. Polymer GARD (P-GARD)	Metabolic-GARD version in which polymers appear.
17. RAF (Reflexively Autocatalytic and Food-Generated)	A measurable and computable version of Kaufmann’s CAS model, similar to GARD.
18. RNA (Replication)-first	One of the core hypotheses in the field of the origin of life, which posits that RNA molecules were the earliest biomacromolecules capable of both genetic inheritance and catalytic functions, serving as the evolutionary precursor to the DNA and protein systems.
19. RNA Replicase	A type of RNA template-dependent nucleic acid polymerase, whose core function is to mediate the replication process of RNA molecules.
20. RNA World	A core hypothesis in the field of the origin of life, proposing that prior to the emergence of the DNA-protein system, there existed a stage of life evolution centered around RNA.
21. SCE—Stable Complex Evolution (Encoding) Model	Stable complexes formed through the folding or accretion interactions of molecules may possess allosteric and chemical transformation capabilities. The molecular configurations that mediate allostery and chemical transformations are considered a form of encoding.
22. Sequence Information	Refers to the specific linear arrangement order of nucleotides in DNA/RNA that encodes the sequence of proteins, which in all constitute genetic instructions guiding the formation of biological structures and functions.
23. Thermodynamic Coding	In the SCE model, energy minimization interactions between molecules encompass specific thermodynamic information.
